# Building capacity to conduct genetic epidemiology research on hyperuricaemia and gout in an Indigenous community in Guam

**DOI:** 10.21203/rs.3.rs-3955100/v1

**Published:** 2024-02-22

**Authors:** Yvette C. Paulino, Frank Camacho, Tristan VC Paulino, Delores J. Lee, Lisa Linda Natividad, Elizabeth Matisoo-Smith, Tony R. Merriman, Anna Gosling

**Affiliations:** University of Guam; University of Guam; University of Otago; University of Guam; University of Guam; University of Otago; University of Birmingham at Alabama - Immunology and Rheumatology Birmingham; University of Otago

**Keywords:** gout, hyperuricemia, pacific islander, genetics, minority health

## Abstract

**Background:**

Gout, the most common inflammatory arthritis disease, and hyperuricaemia onset are influenced by environmental and genetic factors. We sought to investigate these factors in an Indigenous community in Guam.

**Methods:**

In this cross-sectional study, the University of Guam led the qualitative inquiry with the native community, training (pre-screening of participants, data collection methods, and biospecimen handling), study implementation (outreach and recruitment, data collection, and DNA extraction and quantification), and qualitative and epidemiologic data analyses. Recruitment targets were based on demographic representation in current census data. The University of Otago collaborated on ethics guidance, working with Indigenous communities, and led the genetic sequencing and genetic data analysis. Participants were recruited in Guam from Fall 2019 to Spring 2022.

**Results:**

Of the 359 participants, most self-identified as Native CHamorus (61.6%) followed by Other Micronesians (22.0%), and Filipinos (15.6%). The prevalence of metabolic conditions from highest to lowest were obesity (55.6%), hyperuricaemia (36.0%), hypertension (27.8%), gout (23.0%), diabetes (14.9%), cardiovascular disease (8.4%), kidney disease (7.3%), and liver disease (3.4%). Compared to Filipinos and Other Micronesians, significantly more CHamorus had hyperuricaemia (42.1% versus 26.8% in Filipinos and 25.3% in Other Micronesians), gout (28.5% versus 21.4% and 8.9%), diabetes (19.5% versus 8.9% and 6.3%), and hypertension (33.9% versus 19.6% and 16.5%).

**Conclusions:**

We estimated the prevalence of metabolic conditions, especially gout and hyperuricaemia, and found statistical differences among major ethnic groups in Guam, all while obtaining the Indigenous community’s feedback on the genetic study and building gout research capacity. The results of ongoing genetic sequencing will be used to understand molecular causes of gout in Guam.

## Background

Gout is the most common inflammatory arthritic disease worldwide. Inflammation from gout, which occurs in the joints, results from the immune system’s response to monosodium urate (MSU) crystal deposition (1). High concentration of urate (> 6.8 mg/dL) in the bloodstream (hyperuricaemia) is considered essential to the formation of MSU crystals and is the strongest risk factor for gout (2, 3).

A combination of environmental and genetic factors influences both gout and hyperuricemia onset. Consumption of purine-rich foods (e.g. red meat and seafood), tomato, alcohol, and sugar-sweetened beverages are documented to increase gout risk (4–8). Around 183 identified genetic loci affect serum urate concentrations, and 55 also influence gout risk (2, 9, 10). All loci contribute in an additive fashion to a proportion of the variation in urate levels and subsequent risk of gout. Regarding serum urate concentration around 7.7% of serum urate level variation is attributed to identified genetic variation with an additional 20% from genetic variation yet to be identified (2, 10). Currently, identified genetic risk factors of gout and serum urate largely derive from GWAS studies of populations of European ancestry and thus don’t capture additional genetic variation from other populations, especially population-specific variation among underrepresented ethnic minorities (11).

The incorporation of ethnic minority and Indigenous populations such as those from the Pacific Islands in gout genetic research has steadily increased in the last decade. Case-control and cross-sectional studies have identified genetic variants associated with serum urate levels and gout (eg. *SLC22A12, SLC22A11, SLC22A9, ABCC4*) (12–22). Additionally, some studies have found population-specific variants among Pacific Island populations from the South Pacific (eg. ABCC4)(20, 23). Increased prevalence of gout-causing genetic variants among Pacific Island populations may partly contribute to the disproportionate disease prevalence found in Pacific communities with the highest gout prevalence and hyperuricaemia worldwide (24).

Pacific gout research to date has been undertaken almost exclusively among Māori and other Polynesian populations residing in Aotearoa New Zealand or other Polynesian Islands (12, 13, 25–28). Epidemiological studies indicate that the indigenous CHamoru of the Mariana Islands and other Micronesians also suffer from high prevalence of gout and have inherently higher levels of serum urate (29, 30). Archaeological evidence of erosive lesions consistent with gout found in ancient CHamoru meta-tarsal phalangeal joints (most common joints affected by gout) may also indicate that CHamorus has had a long history of gout and hyperuricaemia despite documented changes in dietary and lifestyle factors over time (31, 32). Thus, genetic insight from CHamorus and other Micronesians will provide additional context on how gout impacts all Pacific Island nations and communities.

Various ethical research frameworks have been proposed and developed Field 11, 33, 34 to foster appropriate engagement and empowerment of Indigenous communities in genetic research. Aspects of these ethical frameworks share common principles such as maintaining Indigenous communities’ sovereignty over their genetic data, transparency in data collection, culturally appropriate dissemination of findings, and building capacity to enable communities to participate in the research process. The utilisation of Indigenous-centric research frameworks has been instrumental in gout research among the Māori and other Polynesian populations in Aotearoa New Zealand (11). Therefore, our study is an opportunity to pilot the application of Indigenous-centric research frameworks to communities within Micronesia, emphasising developing the local capacity to conduct genetic research on hyperuricaemia and gout. We aimed to: 1) assess the CHamoru community’s perceptions of gout research, 2) train local researchers on the epidemiologic survey and molecular methods in gout research, 3) test the feasibility of data collection methods and processes, and 4) estimate the prevalence of gout, hyperuricaemia, and related metabolic conditions among the study sample.

## Methods

Figure 1.0 represents the framework for capacity-building to conduct research on hyperuricaemia and gout in Guam. The University of Guam led the qualitative inquiry with the native community, training (pre-screening of participants, data collection methods, and biospecimen handling), study implementation (outreach and recruitment, data collection, and DNA extraction and quantification), and qualitative and epidemiologic data analyses. The University of Otago collaborated on ethics guidance, consultation with Indigenous communities, and led the genetic sequencing and data analysis.

## Seeking input from the CHamoru community

Before the implementation of this hyperuricemia and gout research study, a qualitative inquiry was conducted in June 2019 with self-identified CHamoru residing in Guam and representing various roles including individuals diagnosed with metabolic disease, behavioural health providers, medical health provider, *yo’åmte* or traditional healer apprentice, CHamoru studies/language educators, and Indigenous youth rights. Of the 11 CHamorus interviewed, three were community leaders who participated in key informant interviews while the other eight participated in a focus group session. Content from the hyperuricaemia and gout research study questionnaire was shared with the participants. The qualitative inquiry sought to obtain the CHamoru community’s perceptions of the appropriateness of biological and medical research and the ownership, control, access, and possession OCAP^™^ of health research. OCAPTM^™^ “is the de facto standard for conducting research on First Nations and has grown beyond research to include the governance of all First Nations information” (33).

Key Informant interviews of CHamoru community leaders and focus group discussion data were analysed using traditional thematic analysis. Data codes were developed based on the initial reading of the text, giving rise to specific themes. The CHamorus who participated in the qualitative inquiry felt that the survey questions were appropriate, except for considerable objection to collecting tissue samples like biopsies. Reference was made to historical trauma from the study on amyotrophic lateral sclerosis-parkinsonismdementia complex (ALS-PDC), locally known as lytico-bodig, estimated to be 100 times more prevalent in Guam than in the United States in 1954 (34).

(Unidentified individual): There were researchers trying to establish protocols…would ask for tissue (from) the people who passed away. There were cultural ways of explaining it… researchers never thought out explaining it culturally. They just thought of providing the scientific explanation. More than 70 years later, brain samples collected from people of Guam continues to be used in neurodegenerative disease studies (35) and has “contributed to new ideas for therapies to treat Alzheimer’s”(36). However, blood, urine, and faecal collection may be appropriate when the collection’s purpose and the sample’s use were thoroughly explained to participants.

It was appropriate to “participate in research examining the connection between medical conditions such as diabetes and gout to other Pacific peoples.” Questions related to diet (e.g., sugary foods, fruit, and seafood), use of *åmot* CHamoru or traditional medicine, alcohol use, tobacco use, drug use, height, weight, waist circumference, neck circumference, blood pressure, and disease diagnosis (i.e., high blood pressure, high cholesterol, heart problems, kidney problems, and fatty liver disease) were deemed appropriate. The CHamorus offered caution and considerations when engaging in health research with the native community. For example, while it was appropriate to collect blood samples, the participants felt there might be some resistance and “a thorough explanation would allow for proceeding with this process.” Due to the intrusive nature of blood draws, the CHamorus stipulated that the procedure requires informed consent, literature explaining the research, and ethics approval obtained with Indigenous considerations addressed. These findings were used to inform the research training and data collection.

## Training the research team

The research team included faculty, students, and alumni from the School of Health and the College of Natural and Applied Sciences at the University of Guam. Most of the research team were of Indigenous CHamoru or other Micronesian descent. Team members from the School of Health led the epidemiological support, including survey design, implementation, and data collection consistent with undergraduate student research training (37). The epidemiological support team members had experience in survey implementation and methods in anthropometry collection were standardised. The epidemiological support team received additional training in understanding the goal of the research study, pre-screening and scheduling participant interviews, administering study-specific questionnaires, capturing ancestral history via race and ethnic questions, flagging for referrals participants who indicated hypertension, and handling biospecimen collection. All collected biospecimens were then transferred to the genetic support team. Genetic support included DNA extraction, fluorometric quantification, and quality control. An expert from the University of Otago trained the genetic support team on extraction and quantification protocols and methods.

## Collecting the data

The research team pilot tested the implementation of the hyperuricaemia and gout cross-sectional survey in Guam in Fall 2019. Adult residents of Guam with CHamoru ancestry, at least 18 years old, and able to provide informed consent and come to the designated study site were eligible to participate. Stratified sampling was employed to reflect similar ethnic (CHamoru), gender, and age-group (18 to 30 years old, 40 to 59 years old, 60 years or older) distribution in the 2020 Guam Census. A local laboratory was the designated study site. Individuals were gifted a $20 grocery coupon in appreciation of their generous participation in the study. The research team was able to administer the survey (questionnaires, physical data collection, and biospecimen collection) in a dedicated space within the laboratory. The survey included questions on demographics including maternal and paternal ethnicity, diagnosed metabolic conditions (type 2 diabetes, gout, hypertension, cardiovascular disease, kidney disease, and liver disease), and health behaviours (use of traditional medicine, areca (betel) nut use, alcohol, smoking, exercise, and diet). Physical measurements were collected on height measured in centimetres (Model PE-AIM-101 portable stadiometer, Perspective Enterprises, Portage, Michigan), weight measured in kilograms (Seca 876 portable digital scale, Hamburg, Germany), waist and neck circumferences measured in centimetres (Seca Model 201 plastic tape, Chino, California), and automatic blood pressure measured in millimetres of mercury (Omron, xx). Obesity, represented as body mass index, was determined from height and weight measurements – kilograms/(centimetres*100)2. A body mass index of 30 or higher was classified as obese.

Research participants moved from the survey room to the laboratory, where urine was obtained through self-collection and blood draw was performed by a trained phlebotomist. Urine and blood were used to test for serum urate, haemoglobin A1C (HgbA1c), lipid profile, uric acid, and creatinine. Research participants were immediately shown their anthropometry and blood pressure status. A copy of the laboratory report was offered to the research participants. The team’s research physician reviewed all laboratory reports and provided feedback. Participants were referred to their primary care physician if their measured blood pressure exceeded the hypertensive threshold or if their laboratory results were flagged for values above the reference range thresholds.

A separate 3-mL blood specimen was provided to the research team for DNA extraction at the University of Guam. A Thermofisher MagMax extraction kit was used following the manufacturer’s instructions for blood, with 440 μl of blood utilized per sample. DNA samples were stored in −20°C at the University of Guam while aliquots of samples were sent to the University of Otago for subsequent library preparation for Illumina sequencing. Two levels of sequencing were performed – 30x genomes were generated using Invitrogen Collibri PCR-free ES DNA library preparation kits for Illumina Systems. Low pass sequencing (target of average 4x genome sequencing) used a library preparation method developed by Meyer and Kircher (38). Sequencing was performed on an Illumina NovaSeq 6000 Instrument using an S4 flow cell. Genetic data is housed within University of Otago servers with both the University of Otago and University of Guam investigators with sole access. Data sharing agreements between the University of Otago and the University of Guam will continue to evolve as the data storage and governance capacity at the University of Guam evolves.

## Overcoming recruitment challenges during the pandemic

Experiences gleaned from the pilot test were used to refine the research protocols and processes. Data collection was postponed during the onset of the COVID-19 pandemic but resumed in Fall 2021 and continued through Spring 2022. During this time, recruitment was expanded to include people from other Indigenous Micronesian communities (e.g. Chuuk, Kosrae, Marshall Islands, Palau, Pohnpei, and Yap) and non-Indigenous Filipinos. Recruitment targets for our study were based on sex and ethnic proportions from the latest 2020 Guam census data. Additional protocols and processes were put in place to ensure the safety of the participants and the research team. For example, the data collection team donned personal protective equipment including a mask, face shield, and gloves. The participants were required to wear masks as well. In adherence to social distancing protocols in the laboratory facility, participants provided consent and remained in their vehicles until their appointment.

At the end of recruitment, 359 residents participated in this study. They included CHamorus (n = 221), Other Micronesians (n = 79), Filipinos (n = 56), and Other (n = 3) as shown in Table 1. Other Micronesians included Chuukese, Kosraean, Marshallese, Palauan, Pohnpeian, and Yapese.

## Analysis

R Studio was used to generate the frequencies of demographic factors, behavioural factors, and metabolic disease prevalence using the gtsummary package (39). A Pearson’s Chi-Square was used to determine differences between behavioural factors and metabolic disease prevalence among the predominant ethnic groups (CHamorus, Other Micronesians, and Filipino) as seen in Tables 2.0 and 3.0. A Kruskal-Wallis rank sum test was used to compare mean serum urate between gender (male and female) and ethnicity as seen in Table 4.0.

## Results

As shown in Table 1.0, the 359 participants represented three age categories: 18–39 years (51.0%), 40 to 59 years (31.2%), and 60 years or older (17.8%). Most were females (57%), employed (58.8%), had completed more than 12 years of education (68.6%), and were of CHamoru ancestry (61.6%). Our sample population was slightly skewed in the overrepresentation of females, CHamorus, and Other Micronesians compared to the 2020 Guam census data. The prevalence of metabolic conditions in this study from highest to lowest were obesity (55.6%), hyperuricaemia (36%), hypertension (27.8%), gout (23.0%), diabetes (14.9%), cardiovascular disease (8.4%), kidney disease (7.3%), and liver disease (3.4%) (Table 2.0). Compared to Filipinos and Other Micronesians, statistically significantly more CHamorus had hyperuricaemia (42.1% versus 26.8% in Filipinos and 25.3% in Other Micronesians), gout (28.5% versus 21.4% and 8.9%), diabetes (19.5% versus 8.9% and 6.3%), and hypertension (33.9% versus 19.6% and 16.5%). Behavioural characteristics are presented in Table 3.0 and include alcohol drinking (36.7%), areca (betel) nut chewing (16.9%), engagement in moderate (74.2%) and hard (25.8%) exercise, dietary behaviours (41.6% drank > 1 one sugar-sweetened beverage daily, 47.3% ate > 1 one piece of fruit daily, and 50.2% ate seafood > 1 one day per week), smoking (21.1%), and use of traditional medicine (22.7%). When compared by ethnicity, areca (betel) nut use (35.9%) and daily consumption of > 1 one sugar-sweetened beverage (62.0%) was significantly highest amongst the Other Micronesians subgroup.

Participants were classified as having hyperuricaemia if their serum urate level was ≥ 6.8 milligrams per decilitre. As shown in Table 4.0, mean serum urate was 6.35 mg/dL among CHamorus, 5.65 mg/dL among Filipinos, and 5.90 mg/dL among Other Micronesians. Males had higher mean serum urate levels than females. This pattern is consistent with previous studies indicating sex-specific differences in serum urate like US males (6.35 mg/dL) and females (4.80 mg/dL) in the National Health and Nutrition Examination Survey (NHANES) (40). Similarly, our ethnic-specific serum urate levels were slightly above those reported among ethnic groups in the NHANES such as Non-Hispanic Whites (5.43 mg/dL) and Non-Hispanic African Americans (5.42 mg/dL) (40). Our study’s overall serum urate level is closer to other Pacific Island populations like the Māori from Aotearoa New Zealand (7.1 mg/dL) (41, 42) and American Samoans (6.8 mg/dL) (43). Our reported overall gout prevalence (23.0%) is relatively high within Pacific Island populations such as the New Zealand Māori at 18.4% (42), other Pacific Islanders in New Zealand at 7.6% (44), and Samoans at 7.2% (45). Additionally, our gout prevalence is well above the rest of the United States (5.1%) based on NHANES estimates (46).

The prevalence of hyperuricemia in our study was slightly lower than previous estimates in Guam (30) reported more than 50 years ago (36.0% versus 48% respectively), but the prevalence of gout is considerably higher in this study (23.0% versus 1.5%). The variation in reported gout and hyperuricemia in our study compared to previous Guam reports may be attributed to the convenient sampling approach used in this study as compared to the randomised sampling utilised in previous reporting. However, changes to population structure and lifestyle over the past 50 years in Guam may also influence metabolic disease rates on the island. The transition from an agricultural subsistence lifestyle during the early-mid twentieth century to a diet that incorporates imported processed food items observed in Guam during the twenty- first century may increase the consumption of gout-trigger foods and subsequently influence estimates of gout (32). Given these conditions, the burden of gout may have increased over the past 50 years.

The NHANES is a nationwide survey conducted by the Centre of Disease Control (CDC) which collects dietary, health information, and biochemical data to assess the overall health status of the US; however, US territories such as Guam are not incorporated in this survey (47). Other public health surveillance surveys by the CDC that assess US territories like the Behavioural Risk Factor Surveillance System (BRFSS) do not collect gout data, but it allows states and territories to add additional questions for region-specific health concerns (48). Given the burden of gout and hyperuricaemia observed in our study, similar questions have been added to the annual Guam BRFSS and the first Guam National Health Interview Survey. Though data collection in these surveys is ongoing, this approach offers a cost-effective means for other US territories or states with large Pacific Islander communities to assess the burden of gout.

## Conclusion

In closing, we increased the capacity to conduct genetic research on hyperuricaemia and gout in Guam. Specifically, we found that the Indigenous CHamoru community was receptive to metabolic and related genetic research so long as compliance with ethical standards and representation from native researchers were maintained. We trained Indigenous researchers on epidemiologic surveys and molecular methods and surveyed 359 participants successfully and safely during the COVID-19 pandemic. Finally, we estimated the prevalence of metabolic conditions, especially gout (23%) and hyperuricaemia (51%) and found differences among major ethnic groups in Guam. Genetic sequencing is ongoing, and the results will be used to provide insights into the causes of metabolic disease in Guam.

## Figures and Tables

**Figure 1:**
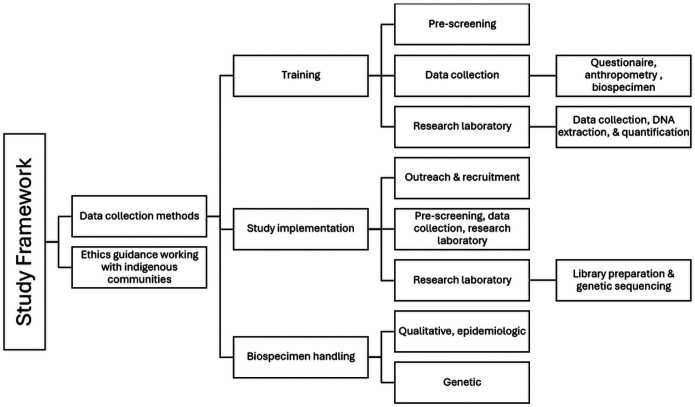
Figure 1.0 Capacity framework for metabolic disease research

**Table 1.0:** Demographic characteristics of the study sample

Characteristic	n = 359^1^
Age (years)	
18 to 39	183 (51.0%)
40 to 59	112 (31.2%)
65 or older	64 (17.8%)
Gender	
Female	204 (57.0%)
Male	153 (42.7%)
Transgender	1 (0.3%)
Ethnicity	
CHamoru	221 (61.6%)
Filipino	56 (1 5.6%)
Other	3 (0.8%)
Other Micronesian	79 (22.0%)
Education	
12 years	72 (20.3%)
Less than 12 years	39 (11.0%)
more than 12 years	243 (68.6%)
Employment	
Employed	211 (58.8%)
Other	20 (5.6%)
Retired	40 (11.1%)
Student	53 (14.8%)
Unemployed	35 (9.7%)

1 n (%)

Due to missing data, some frequencies (Gender, Ethnicity, and Employment) may not equal 359.

**Table 2.0:** Metabolic conditions

Characteristic	Overall, n = 356^1^	CHamoru, n = 221^1^	95% CI^2^	Filipino, n = 56^1^	95% CI^2^	Other Micronesian, n = 79^1^	95% CI^2^	P value^3^
Cardiovascular Disease	30 (8.4%)	20 (9.0%)	5.8%, 14%	2 (3.6%)	0.62%, 13%	8 (10.1%)	4.8%, 19%	0.361
Diabetes	53 (14.9%)	43 (19.5%)	1 5%, 25%	5 (8.9%)	3.3%, 20%	5 (6.3%)	2.4%, 15%	0.008*
Gout	82 (23.0%)	63 (28.5%)	23%, 35%	12 (21.4%)	12%, 35%	7 (8.9%)	3.9%, 18%	0.002*
Hypertension	99 (27.8%)	75 (33.9%)	28%, 41%	11 (19.6%)	11 %, 33%	13 (16.5%)	9.4%, 27%	0.004*
Chronic Kidney Disease	26 (7.3%)	21 (9.5%)	6.1%, 14%	3 (5.4%)	1.4%, 16%	2 (2.5%)	0.44%, 9.7%	0.103
Liver Disease	12 (3.4%)	11 (5.0%)	2.6%, 9.0%	1 (1.8%)	0.09%, 11%	0 (0.0%)	0.00%, 5.8%	0.092
Obesity	198 (55.6%)	133 (60.2%)	53%, 67%	17 (30.4%)	19%, 44%	48 (60.8%)	49%, 71%	< 0.001*
Hyperuricemia	128 (36.0%)	93 (42.1%)	36%, 49%	15 (26.8%)	1 6%, 41%	20 (25.3%)	16%, 37%	0.009

1 n (%).

2 95% Confidence interval

3 Pearson χ2 test

* Significant (P < 0.05)

Hyperuricemia is defined as participants with serum urate > 6.8 mg/dL. Obesity was defined as having a bmi ≥ 30 kg/cm^3^.

**Table 3.0:** Behavioural Factors

Characteristic	Overall, N = 356^1^	CHamoru, N = 221^1^	95% CI^2^	Filipino, N = 56^1^	95% CI^2^	Other Micronesian, N = 79^1^	95% CI^2^	P value^3^
Alcohol Consumption	128 (36.7%)	87 (39.7%)	33%, 47%	20 (37.0%)	25%, 51%	21 (27.6%)	18%, 39%	0.169
Betel Nut Use	34 (16.9%)	6 (8.8%)	3.6%, 19%	0 (0.0%)	0.00%, 8.1%	28 (35.9%)	26%, 48%	< 0.001*
Exercise								0.685
moderate	264 (74.2%)	165 (74.7%)	68%, 80%	39 (69.6%)	56%, 81%	60 (75.9%)	65%, 85%	
regular hard PA	92 (25.8%)	56 (25.3%)	20%, 32%	17 (30.4%)	19%, 44%	19 (24.1%)	1 5%, 35%	
Fruit Consumption								0.352
more than one	166 (47.3%)	98 (44.3%)	38%, 51%	29 (52.7%)	39%, 66%	39 (52.0%)	40%, 64%	
one or less	185 (52.7%)	123 (55.7%)	49%, 62%	26 (47.3%)	34%, 61%	36 (48.0%)	36%, 60%	
Sea Food Consumption								0.230
more than one	166 (50.2%)	99 (46.7%)	40%, 54%	30 (54.5%)	41 %, 68%	37 (57.8%)	45%, 70%	
one or less	165 (49.8%)	113 (53.3%)	46%, 60%	25 (45.5%)	32%, 59%	27 (42.2%)	30%, 55%	
Smoking	75 (21.1%)	51 (23.1%)	18%, 29%	9 (16.1%)	8.1%, 29%	1 5 (19.0%)	11 %, 30%	0.453
Sugar-Sweetened Beverage Use								< 0.001*
more than one	148 (41.6%)	81 (36.7%)	30%, 43%	18 (32.1%)	21 %, 46%	49 (62.0%)	50%, 73%	
one or less	208 (58.4%)	140 (63.3%)	57%, 70%	38 367.9%)	54%, 79%	30 (38.0%)	27%, 50%	
Traditional Medicine (Amot)	80 (22.7%)	47 (21.3%)	16%, 27%	9 (16.7%)	8.4%, 30%	24 (30.8%)	21 %, 42%	0.118

1 n (%).

2 95% Confidence interval

3 Pearson χ2 test

* Significant (P < 0.05)

**Table 4.0:** Serum urate by sex and ethnicity

Characteristic	Overall, n = 354^1^	Female, n = 203^1^	Male, n = 151^1^	P value^2^
Sex	-	5.60 (5.4, 5.8)	7.47 (7.2, 7.8)	< 0.001*
Ethnicity				< 0.095
CHamoru	6.35 (5.10, 7.77)	5.40 (4.50, 6.45)	7.30 (6.35, 8.50)	
Filipino	5.65 (5.00, 6.80)	5.50 (4.70, 5.90)	7.70 (6.10, 9.30)	
Other Micronesian	5.90 (5.32, 6.80)	5.75 (4.97, 6.43)	6.75 (5.82, 8.08)	

1 mean (mg/dL) (95% confidence interval)

2 Kruskal-Wallis rank sum test

* Significant (P < 0.05)

## Data Availability

Participants of this study did not provide written consent for data to be publicly shared. Code used to generate tables using the gtsummary package can be accessed: https://github.com/paulinot7400/Building-Capacity---Guam-Manuscript.git.
